# Microtubules and mechanosensing: key players in endothelial responses to mechanical stimuli

**DOI:** 10.1007/s00018-025-05828-0

**Published:** 2025-08-21

**Authors:** Danahe Mohammed, Ibrahim Hamid, Benoit Vanhollebeke, Maud Martin

**Affiliations:** 1 Laboratory of Neurovascular Signaling, Department of Molecular Biology, ULB Neuroscience Institute, Université Libre de Bruxelles (ULB), Gosselies, Belgium; 2 WEL Research Institute (WELBIO Department), Wavre, Belgium; 3 Center for Microscopy and Molecular Imaging, Université Libre de Bruxelles (ULB), Gosselies, Belgium

## Abstract

The vascular mechanical microenvironment is characterized by dynamic forces such as blood flow, stretch, and matrix stiffness, which profoundly influence endothelial cell (EC) behavior. ECs detect these forces through specialized mechanosensing structures and activate mechanotransduction pathways to adapt their responses and maintain vascular homeostasis. While actin filaments and focal adhesions are well-established mediators of these processes, emerging evidence highlights microtubules as critical players in endothelial mechanotransduction. Composed of α- and β-tubulin, microtubules are stiff elements forming a dynamic and adjustable network that regulates cell polarity, migration, and signaling. Their characteristics make them interesting candidates as essential regulators in force sensing, modulating cellular stiffness and adaptation to mechanical constraints. In this Review, we discuss the role of microtubules in endothelial mechanosensing, emphasizing their contribution to force perception and cellular adaptation. Specifically, we describe their involvement in shear stress sensing, curvature and matrix stiffness detection, pressure response, and topographical sensing. Furthermore, we highlight how microtubules are dynamically modified upon mechanical cues and explore the role of post-translational modifications, particularly acetylation, in regulating their mechanical properties. These insights provide a new perspective on endothelial responses to mechanical stimuli, offering potential therapeutic avenues in the context of pathological angiogenesis, where microtubule regulation may play a crucial role.

## Introduction

**Fig. 1:**
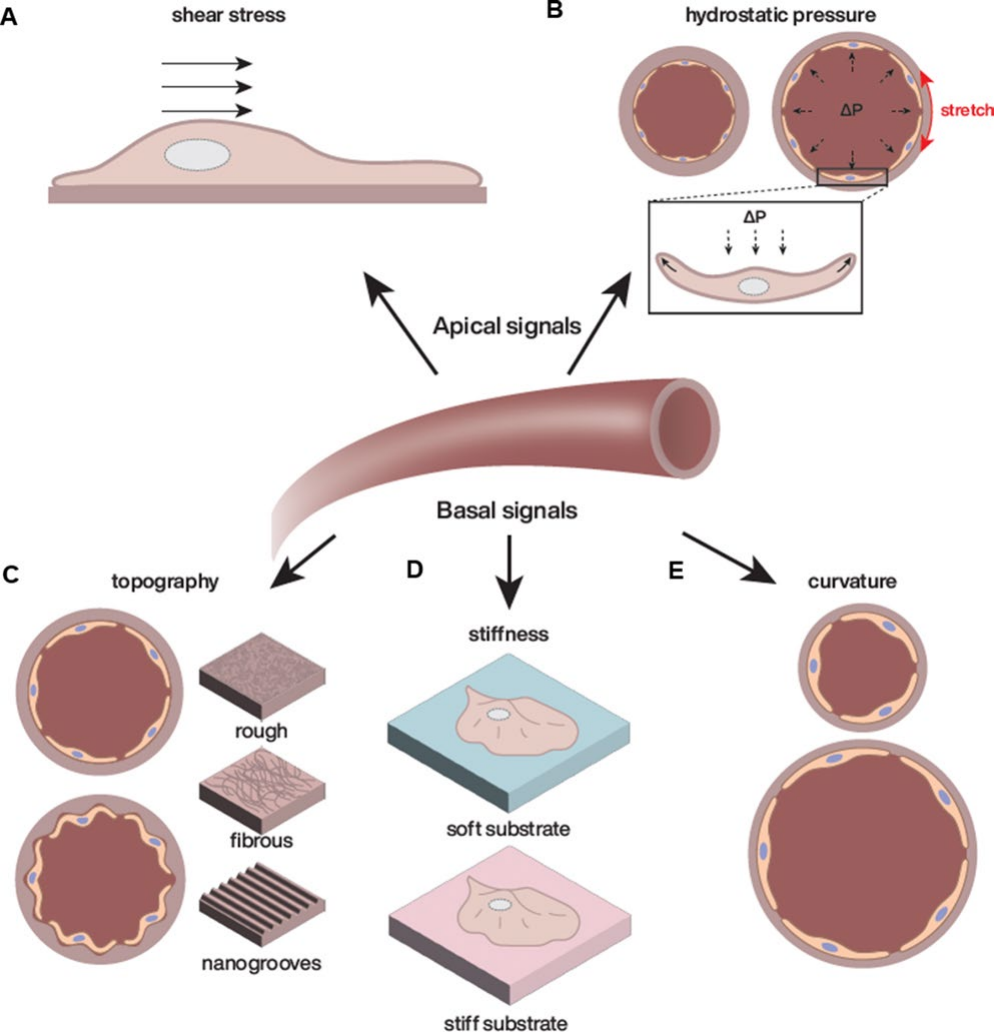
Illustration of the various mechanical constraints influencing cells within their microenvironment. (**A**) Shear stress exerted on cells by fluid flow. (**B**) Compressive stress induced by the blood hydrostatic pressure (dashed black arrows) and consequent circumferential stretch due to vessel dilation (red arrow). (**C**) Substrate topography impact on cell behavior. (**D**) Substrate stiffness sensed by the cell. (**E**) Effect of substrate curvature on cellular responses

Extensive research over the last twenty years has demonstrated that mechanical forces play a crucial role in shaping cellular architecture and function in both physiological and pathological contexts [1–3]. These mechanical stresses are especially pronounced in the vascular system, where endothelial cells (ECs) are subjected to dynamic forces induced by circulating blood [3–7]. These apical forces include shear stress [3, 4] resulting from the friction of blood flow against the vessel wall, hydrostatic pressure [7, 8] exerted by the column of blood on the endothelial surface and circumferential stretch [9] caused by pulsatile pressure leading to vessel dilation. ECs have evolved highly specialized mechanosensory mechanisms to detect and respond to these forces, ultimately regulating vascular homeostasis. The basal side of ECs is also subjected to physical forces: the endothelial microenvironment is characterized by a complex interplay of mechanical stresses with a specific topography [10, 11] and varying stiffness [12, 13] depending on the type of blood vessel. The curvature of the vessels, which depends on their diameters, imposes another spatial constraint [14]. The ability of ECs to integrate these different biophysical cues (Fig. 1) is essential for orchestrating vascular function and adapting to physiological and pathological conditions.

Mechanotransduction relies on various mechanosensors that detect and convert mechanical stimuli into biochemical responses. Among them, the mechanosensitive ion channels Piezo1/2 respond to mechanical forces by modulating ion influx [15]. Integrins, associated with mechanosensitive adaptor proteins like talin and vinculin, anchor cells to the extracellular matrix and activate intracellular signaling [16]. The complex comprising PECAM-1 (Platelet EC Adhesion Molecule-1), VE-Cadherin (Vascular Endothelial-Cadherin) and VEGFR2 (Vascular Endothelial Growth Factor Receptor 2), found at endothelial junctions, transduces intercellular tension into intracellular signals [3]. Other important mechanosensors include the glycocalyx, a layer of polysaccharides and glycoproteins covering the cell membrane. One of its components, hyaluronic acid (HA) and its main receptor CD44, are particularly important in responding to mechanical forces from blood flow and are stabilized by the spectrin network that senses membrane tension [17]. G protein-coupled receptors (GPCRs), such as angiotensin receptors and lysophosphatidic acid receptors, can also act as mechanosensors by responding to mechanical forces and activating downstream signaling pathways [18]. The LINC (Linker of Nucleoskeleton and Cytoskeleton) complex that links the cytoskeleton to the nucleus, and regulatory proteins like the transcriptional regulators YAP/TAZ, whose location and activity are dictated by mechanical forces, contribute to mechanotransduction by modulating gene expression [19].

**Fig. 2:**
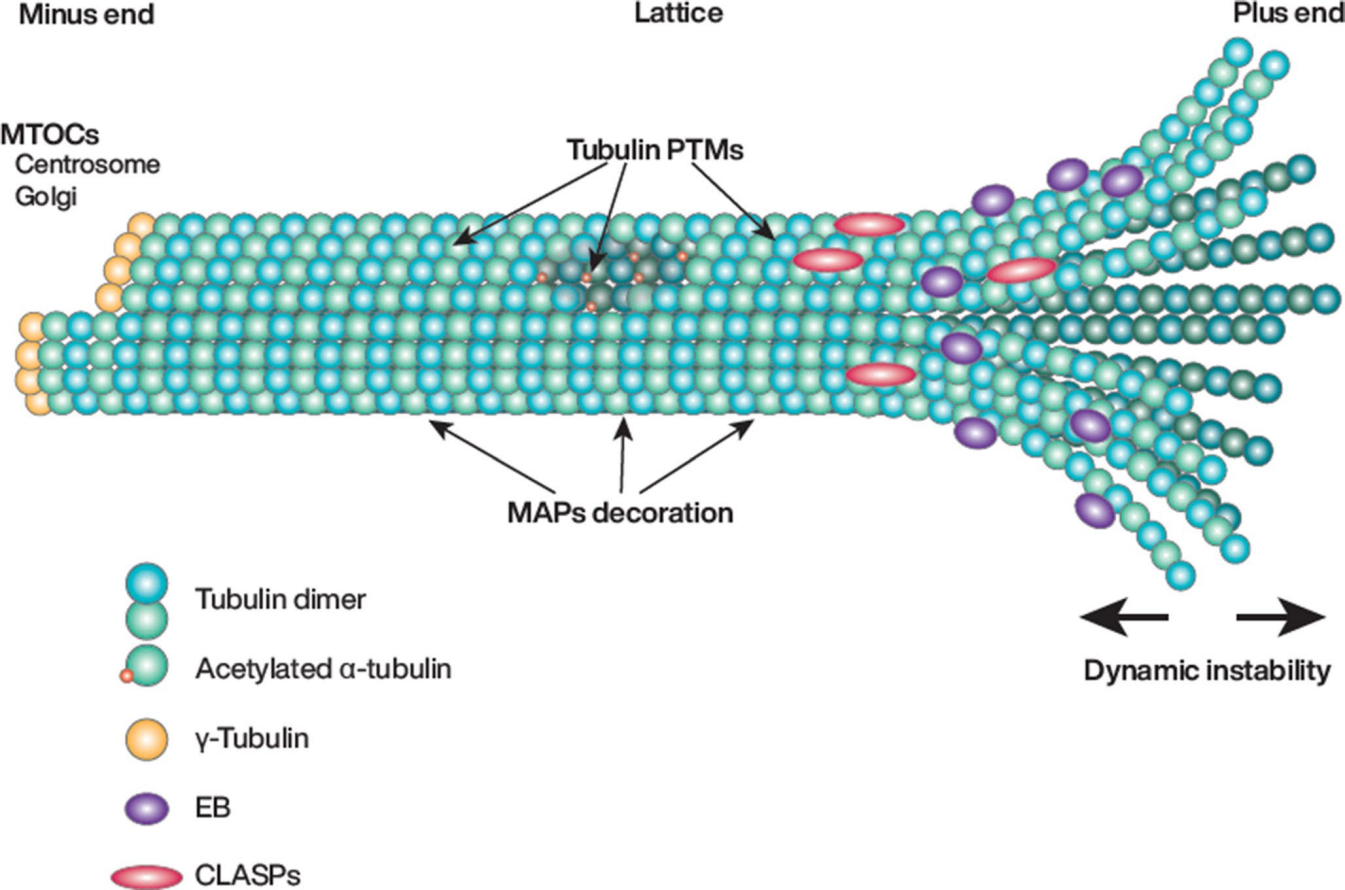
Schematic representation of the structural and functional zones of a microtubule. The microtubule is composed of tubulin dimers (blue and green spheres) organized into a cylindrical lattice. From this central region, two distinct ends are extending. The plus-end is the dynamic growing end where polymerization predominantly occurs and is submitted to dynamic instability. The minus-end is the stabilized extremity that is often anchored at microtubule-organizing centers (MTOCs), impacting on microtubule geometrical organization. Microtubule-associated proteins (MAPs) bind along the lattice, as well as on the plus- and minus-ends, regulating various microtubule properties. In addition, various post-translational modifications (PTMs), such as acetylation and detyrosination, are distributed along the lattice, influencing microtubule stability and interactions with associated proteins

While the actin cytoskeleton and focal adhesions have long been recognized as primary actors in mechanosensing [20–22] the contribution of microtubules in endothelial mechano-transduction remains poorly understood, although emerging evidence suggests that microtubules play a critical role in sensing and adapting to mechanical forces [23–25]. Microtubules are key cytoskeletal components that provide structural support and hold key functions in intracellular transport [26] cell division [27] and signal transduction [28]. Composed of α/β-tubulin heterodimers, microtubules dynamically assemble and disassemble through a process known as dynamic instability, regulated by GTP hydrolysis [29]. This intrinsic behavior enables cells to rapidly reorganize their cytoskeleton in response to environmental cues, including mechanical forces [30]. Adding to this adaptability, microtubules form diverse populations, carrying functional heterogeneity, whose attributes can be actively modified [31] (Fig. 2). A wide range of microtubule-associated proteins or MAPs, dynamically interacts with microtubule filaments at different locations, impacting their anchoring, organization, stability, assembly, and dynamics [32, 33]. Tubulin undergoes various post-translational modifications (PTMs), including common ones like phosphorylation, acetylation, and ubiquitylation, as well as tubulin-specific modifications such as detyrosination, glutamylation or glycylation, which are thought to dictate specific properties [31]. Among these, acetylation of α-tubulin at Lys40 stands out as a prominent modification in response to various mechanical cues.

An important property of the microtubule network to consider is its stability. However, the term should be used with care, as stability can refer to distinct aspects of microtubule behavior. In most cases, stable microtubules relate to filaments that have increased structural stability making them more resistant to complete depolymerization under stress. This form of stability is not directly related to rigidity or stiffness but is instead associated with microtubule longevity or “age”. Microtubule dynamics (often referred to as dynamic instability) is a different process and describes the peculiar behavior of microtubule ends, which undergo rapid transitions between phases of growth and shrinkage. Preventing or slowing down these dynamics of microtubule ends could also be viewed as stabilizing them and is sometimes called hyper stabilization. As long-lived microtubules have time to accumulate PTMs, microtubules that are stable overtime are often acetylated, leading to the assumption that acetylation stabilizes microtubules. It has indeed been recently shown that acetylated microtubules have decreased flexural rigidity, making them more resistant to bending-induced breakage [34–36] supporting a model in which acetylation enhances microtubule lattice pliability and promotes their capacity to deform without mechanical breakage, thereby extending their lifetime under mechanically challenging conditions. Nevertheless, the causality between this modification and microtubule stability in cells remains elusive and warrants further investigation.

Microtubule network organization, which plays a crucial role in regulating cell architecture, strongly depends on the distribution of the sites responsible for microtubule minus-end attachment. These sites are not always located at the centrosome [37] but can also include the Golgi apparatus, as seen in ECs [38]. Many if not all these microtubule properties are interconnected and can influence one another, yet their precise relationships remain largely unknown.

Microtubules have been known for a long time to be essential for the formation of angiogenic structures but the precise role of specific microtubules during angiogenesis is only emerging [38–40]. Non-centrosomal microtubules, stabilized by CAMSAP2, enable ECs to establish polarity and directional migration. Depletion of non-centrosomal microtubules leads to unstable protrusions, impairing sprout elongation in 3D models and causing defective vascular development in vivo [38]. Detyrosinated microtubules specifically control venous sprouting during lymphovenous development in zebrafish [40]. Beyond their role in EC sprouting, microtubules have been recently identified as critical regulators of the endothelial junction stability which is required for proper blood vessel formation [39].

While previous reviews have mainly addressed the role of the actin cytoskeleton and integrin-based adhesions in endothelial mechanosensing, a comprehensive survey focusing specifically on microtubules remains lacking. Our review integrates recent advances in microtubule biology across a broad spectrum of mechanical stimuli that were rarely analyzed collectively, including shear stress, substrate stiffness, stretch, curvature, and topography. It notably emphasizes the emerging role of microtubule PTMs in EC responses to mechanical stress. By connecting microtubule modifications to signaling pathways and to cellular mechano-adaptation, this review provides a new perspective on endothelial responses to mechanical environments, complementing existing models dominated by actin-centered paradigms.

We first describe how the microtubule network is modified in response to mechanical stress, identifying the upstream mechanosensors as well as downstream molecular and cellular effectors. We then examine key features of each environmental constraint and discuss how microtubules modulate their effect on EC morphology, intracellular organization, and overall function. Where relevant, we incorporate key observations from other cell types that may offer insights applicable to the endothelium. Finally, we highlight open questions and discuss how a deeper understanding of these microtubule-associated mechanisms could enhance our knowledge of EC responses to complex vascular environments.

## Microtubule modifications under mechanical stress

**Fig. 3:**
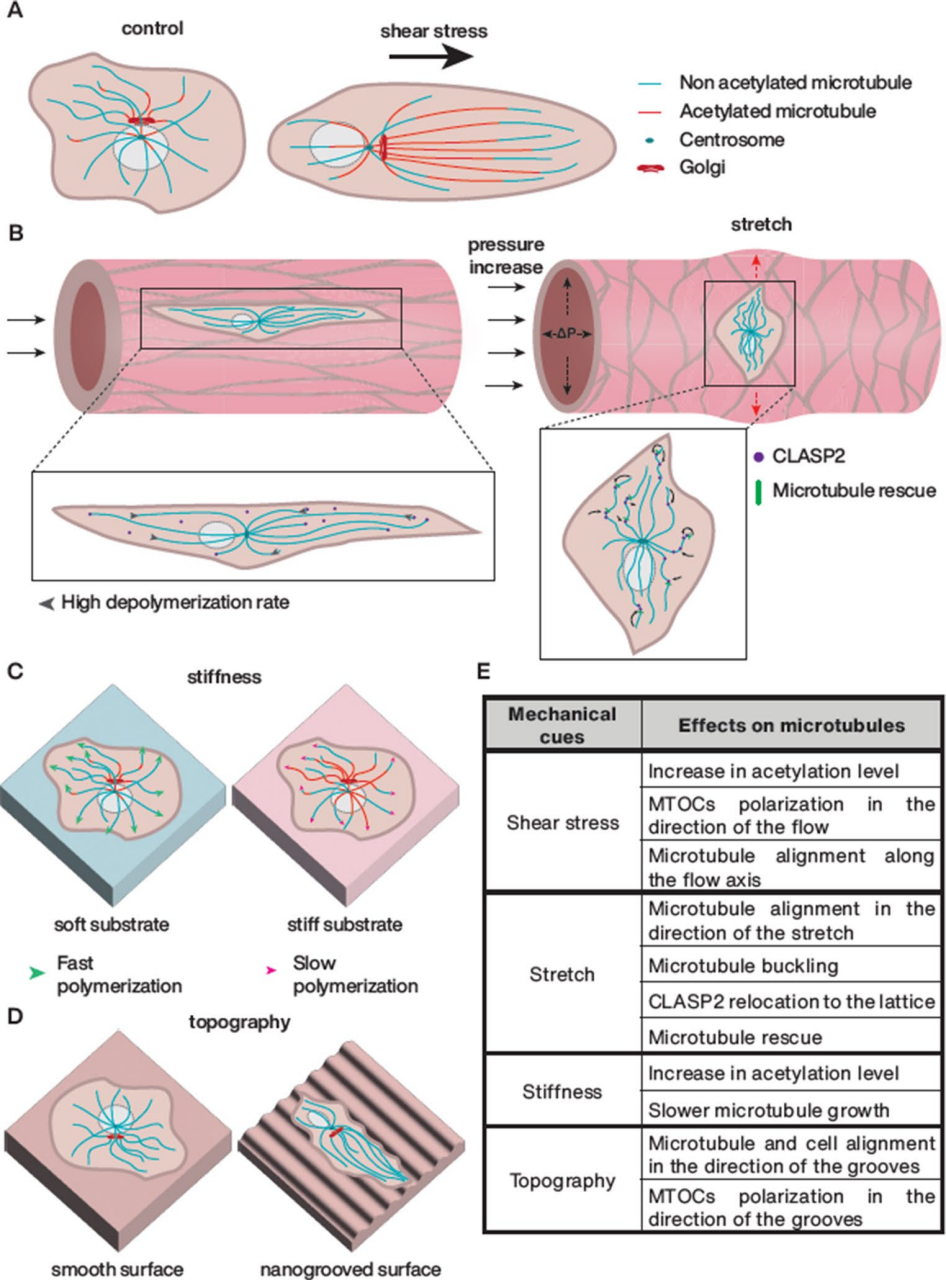
Effects of mechanical constraints on microtubule dynamics and cell polarization. (**A**) Shear stress leads to increased microtubule acetylation, cell polarization in the direction of flow, and cell elongation along the flow axis. (**B**) Axial stretch is associated with alignment of cell and microtubules in the direction of stretch, microtubule buckling, CLASP2 relocalization from microtubule plus-ends to curved sites as indicated by the small arrows, and decreased microtubule depolymerization. (**C**) Stiffness induces microtubule hyperacetylation and decreased polymerization rate. (**D**) Cells align and polarize along the nanogrooved topography. (**E**) Table summarizing microtubule modifications upon mechanical stresses

Microtubules dynamically respond to various mechanical stresses, with their integrity, organization, dynamics, and PTMs varying depending on the type of stress encountered (Fig. 3). Under fluid shear stress, microtubules align with the direction of the flow [25, 41]. This mechanical cue not only reorganizes the cytoskeleton but also increases microtubule acetylation, a postulated marker of stabilized and long-lived microtubules [25]. Shear stress has also been documented to regulate intracellular polarization: under high shear stress and VEGF-A stimulation, the Golgi and centrosome position themselves against the flow, whereas under low shear stress, they align with the flow and remain randomly distributed under static conditions [42]. This polarization, which directs cellular responses as migration, directly influences microtubule organization since microtubules are primarily anchored at the Golgi or the centrosome in ECs. The ability of microtubules to build an asymmetrical network [43] has been shown to be crucial for supporting EC polarization during sprouting angiogenesis, especially through the non-centrosomal population [38]. In the context of mechanical stretching associated with blood vessel dilatation, microtubules align along the stretch axis [44] become less dynamic and their lattice becomes decorated with CLASP proteins, which stabilizes the microtubule lattice against depolymerization [24]. Mechanical stress at the basal side also affects microtubule properties. Increased substrate stiffness enhances microtubule acetylation [23] and alters their dynamics, with higher microtubule growth speed observed on soft substrates [45]. Moreover, on aligned topographies, microtubules orient themselves along the patterned lines, promoting cell elongation [46].

Overall, microtubules actively respond to the mechanical cues of their environment by adapting their organization, often aligning their network with the direction of the force, modifying their PTM levels, particularly acetylation, increasing their stability against depolymerization, and regulating their plus-end dynamics. These modulations, which can be interrelated, likely reflect an intrinsic role for microtubules in mechanosensing.

## Upstream regulators of microtubule modifications

**Fig. 4:**
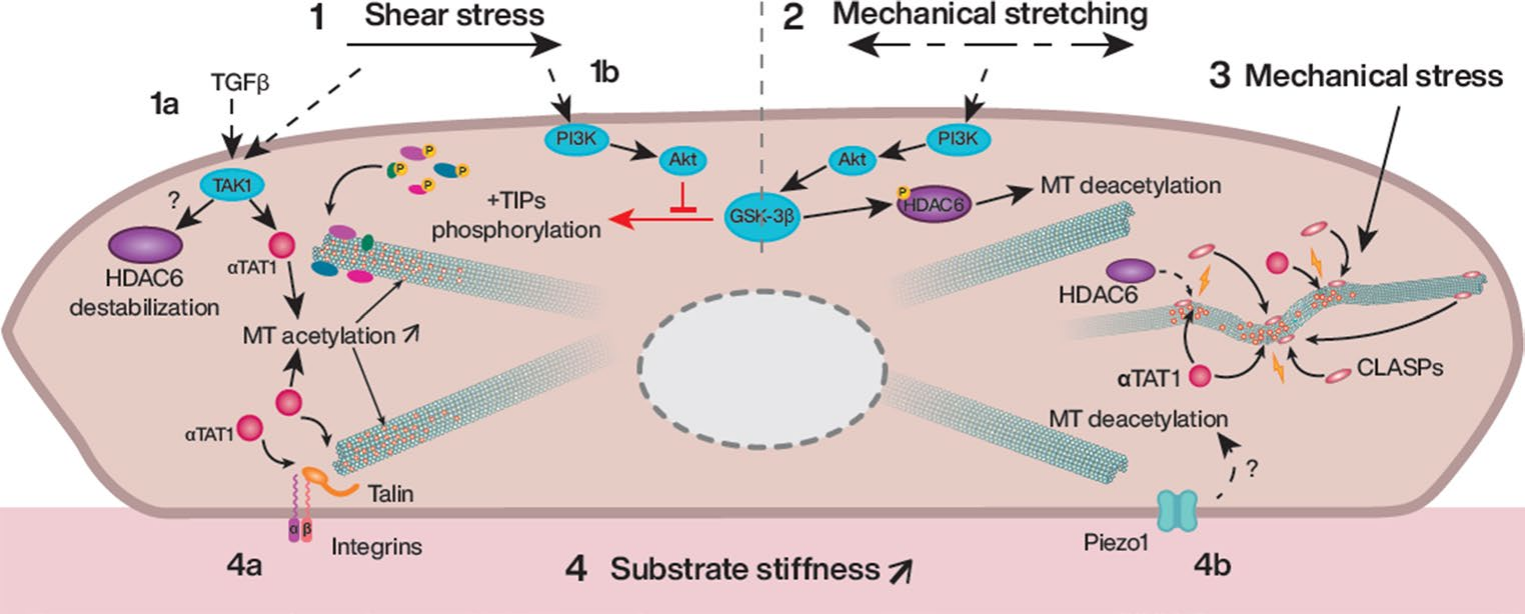
Schematic representation of upstream regulators of microtubule modifications in the context of mechanical constraints. (1a) Shear stress acts on TAK1 which activates α-TAT1, inducing microtubule hyperacetylation. Shear stress has also been associated with HDAC6 destabilization; (1b) Shear stress activates PI3K/Akt signaling, inhibiting the phosphorylation of plus-ends tracking proteins by GSK-3β, thereby allowing their attachment to microtubule plus-ends, where they regulate microtubule growth and crosslink with actin; (2) Mechanical stretching response might also involve PI3K/Akt signaling which activates GSK-3β, which then phosphorylate and activate HDAC6 leading to microtubule deacetylation; (3) Mechanical forces have a direct effect on microtubules, making them buckle upon compression and damaging them. Physical damages permit entry of α-TAT1 and HDAC6 through the cracks, resulting to both acetylation then deacetylation with different kinetics. These events also lead to the recruitment of CLASP, stabilizing the lattice and protecting it from catastrophe; (4a) In response to substrate stiffening and β1 integrin activation, talin accumulates α-TAT1 at focal adhesions, promoting microtubule acetylation. (4b) In neural crest cells, the mechanosensitive ion channel Piezo1 drives microtubule deacetylation upon substrate stiffening, through an unknown signaling pathway

Microtubules undergo fine modifications in response to various mechanical constraints. These alterations are likely mostly mediated by specific signaling pathways initiated by membrane mechanoreceptors that convert physical forces into intracellular biochemical signals [47] (Fig. 4). An important cytosolic messenger seems to be glycogen synthase kinase-3β (GSK-3β), a kinase that phosphorylates many microtubule plus-ends tracking proteins, such as EB, CLASP, APC and ACF7, detaching them from the filaments and thereby dysregulating microtubule growth and crosslinking with actin [48]. GSK-3β is phosphorylated in response to blood flow, a modification that inhibits its activity [41]. At the same time, the presence of a GSK-3β inhibitor or the expression of a constitutively active form of the kinase alters the flow-induced cell elongation and reverses centrosome polarization, indicating that tight regulation of GSK-3β activity is critical for endothelial morphological responses to shear [41]. GSK-3β is known to negatively respond to PI3K/Akt signaling, a pathway well-described to be activated in this context [49]. Mechanical stretching influences GSK-3β in an opposite way. It promotes its activity which in turn phosphorylates and activates HDAC6, leading to α-tubulin deacetylation. GSK-3β pharmacological blockade or cAMP/EPAC signaling-induced inhibition of HDAC6 counteracts the endothelial hyperpermeability produced by stretching [50]. PI3K/Akt signaling may also be involved in this stretching context, as inhibition of this pathway has been associated with GSK-3β activation in stretched skeletal muscle [51] suggesting that PI3K/Akt may be oppositely regulated during shear stress versus mechanical stretch. In both cases, GSK3-β activity is inversely correlated with microtubule acetylation in endothelial cells. However, the regulatory mechanisms, impacting on HDAC6 activity in the two scenarios, appear to differ: shear stress destabilizes the protein levels, whereas stretching modulates its activating phosphorylation [25, 50]. These findings highlight the critical influence of the mechanical environment on signaling pathways and cellular responses.

Pointing to a key role for this modification, the main enzymes that control microtubule acetylation status (α-TAT1 and HDAC6) emerge as important mediators of microtubule-associated mechanosensing. Upon activation of β1 integrins in response to increased substrate rigidity, talin, a key mechanosensitive integrin partner, recruits and concentrates α-TAT1 at focal adhesions, enhancing microtubule acetylation [23]. Another type of mechanoreceptor, the mechanosensitive ion channel Piezo1, mediates microtubule deacetylation in neural crest cells to adjust cellular mechanics when the substrate on which cells collectively migrate becomes stiffer, although the exact molecular signaling and its potential conservation in ECs remain unknown [52]. In the context of shear stress, inhibition of TAK1, a TGF-β-activated kinase that has been shown to activate α-TAT1 and induce hyperacetylation of microtubules [53] specifically blocks flow-induced acetylation, but not the basal level [25]. Given the importance of TGF-β signaling in flow sensing [54, 55] these observations could molecularly and functionally link this pathway to microtubule acetylation and the EC shape response.

A common intracellular messenger in mechanosensing is the transient and localized increase in intracellular calcium, which can contribute to the regulation of microtubule organization and properties [56, 57]. In vitro, an increased concentration of Ca^2+^ enhances the microtubule depolymerization rate and frequency [58] suggesting that this control may operate in a direct manner.

In addition to these signaling pathways, microtubules themselves may function as mechanosensors, directly responding to mechanical tension. In vitro experiments suggest that microtubules may have specific mechanoresponsive properties: they align along tensile stress, buckle upon compression and grow faster under tension [59, 60]. Physically-damaged microtubules by laser ablation or bending forces can trigger incorporation of new tubulin dimers within the microtubule lattice [61] an event called self-repair that was shown to make microtubules more resistant to disassembly [62].

In plant cells, cortical microtubules self-align with stress patterns [63] whereas microtubule self-repair has been demonstrated in animal cells [64]. In addition to transmitting mechanical information, it is therefore possible that microtubules directly perceive mechanical signals, transferred by cell membrane tension or deformation for instance, and change their spontaneous behavior. The physical constraints could alter the conformation of microtubules, curve or damage them, and these modifications could impact microtubule dynamics, either directly by affecting thermodynamic parameters, or indirectly via the specific recruitment of microtubule-associated factors. For instance, CLASP, a MAP that stabilizes damaged lattices, has been shown to localize at bent microtubule regions and to be recruited to defect sites [65]. Similarly, acetylation decorates highly curved microtubules [66] and damage sites can offer efficient entry sites for microtubule modifying enzymes such as α-TAT1 or HDAC6 [66, 67]. These structural and chemical changes could collectively serve as mechanosensors and allow microtubules to respond dynamically to mechanical forces.

In the context of microtubule deformation, one can think about primary cilia, microtubule-based structures whose bending has been proposed as sensor of shear stress generated by flow in blood vessels. While the molecular mechanism coupling cilia movement and mechanotransduction is unclear, and as microtubules are exceptionally stable and extensively post-translationally modified in cilia, one hypothesis could rely on the presence of sensors directly connected to microtubules, either a shear sensitive-channel linked to adjacent microtubule doublets that would open in response to the internal shear or a curvature sensor attached to the microtubule doublet in the axoneme and sensing the torque of the cilium [68].

## Cellular consequences of microtubule modifications

Microtubules generate forces through polymerization and depolymerization dynamics [69]. Notably, growing and shrinking microtubule ends can exert forces of ∼0.5 and ∼5 pN, respectively [70]. This ability, combined with their intrinsic rigidity, distinguishes them from actin filaments. As the stiffest cytoskeletal filament, microtubules can withstand substantial forces and bear compressive loads exerted by the surrounding contractile cytoskeleton, although they are susceptible to buckling under extreme compressive loads within cells [71]. As such, microtubules can directly mechanically impact cell shape and behavior. This is particularly relevant because the microtubule modifications observed in response to forces can dynamically regulate their mechanical properties. For instance, recruitment of MAPs, such as Tau, can impact microtubule stabilization and bundling, thereby enhancing their resistance to rupture [72]. PTMs of microtubules also modify their physical characteristics. As described before, microtubule acetylation can reinforce their mechanical stability [36] and regulate cellular mechanics by increasing cell stiffness [52].

Microtubules also mediate force transmission between the cytoplasm and the nucleus via the LINC complex. This complex, composed of Nesprin and KASH proteins in the outer nuclear membrane and of SUN proteins in the inner nuclear membrane, establishes a direct mechanical link between the cytoskeleton and the nucleus [73, 74]. Microtubules interact with the LINC complex through molecular motors facilitating the transfer of mechanical forces exerted on the cytoskeleton [75, 76]. This force transmission, described by the cellular tensegrity theory [77] leads to nuclear deformations, influencing chromatin organization and gene expression regulation [78, 79]. While the classical model describes microtubules as transmitters of signals toward the nucleus, recent findings reveal an unexpected inverse mechanism, wherein the nucleus can impact the cell periphery via microtubules. Specifically, the nuclear envelope protein SUN1, stabilizes peripheral microtubules and their interactions with cell junctions, a long-range nuclear communication pathway that is crucial for endothelial junction stability [39].

In addition to their structural role, microtubules can exert regulatory functions through their binding with other cellular components, interactions that can be dynamically modulated [80, 81]. The associations between microtubules and focal adhesions [82] as well as with actin filaments [83] are central to mechanotransduction, enabling cells to sense and respond to external forces. The microtubule-actin crosstalk facilitates the transmission of mechanical cues across the cell, regulating adhesion dynamics and cellular tension [23, 84]. Beyond their physical interaction, microtubules are well known to regulate the actin cytoskeleton and its associated contractility by modulating the localization and activity of GEF-H1, a Rho GEF (guanine nucleotide exchange factor) that is sequestered in its inactive state on microtubules [85]. Upon depolymerization or acetylation, microtubules release GEF-H1 into the cytosol, leading to the activation of RhoA, a small GTPase that enhances actomyosin contractility via ROCK signaling. This process strengthens the traction forces essential for mechanosensitive responses, including adhesion and migration [23]. Of note, microtubule depolymerization using nocodazole, a widely used experimental approach to assess the role of microtubules, induces cell hyper contractility by this mechanism.

The observation that microtubule ends are often found in the vicinity of focal adhesions has led to the discovery of specific regulatory mechanisms that direct microtubule towards focal adhesions to regulate their dynamics. Regulators of this process include specialized cortical complexes that capture microtubules via MAPs [86, 87] microtubule-focal adhesion crosslinkers [88] and actin-binding proteins [20]. Inhibiting any of these components disrupts proper microtubule recruitment to focal adhesions, resulting in excessive adhesion stability and impaired mechanotransduction. Additionally, microtubule acetylation has been shown to participate in this process that promotes focal adhesion turnover by enhancing Rab6-positive vesicle fusion at adhesion sites [89] and by increasing cell contractility as described above [23].

An important function of microtubules that can also influence cell behavior is the intracellular transport of organelles, vesicles, and proteins. While the intracellular roads that these filaments build will clearly be impacted by microtubule stability and organization, microtubule PTMs also influence their interaction with kinesin and dynein molecular motors, constituting a regulatory code for intracellular transport [31, 90]. Among these modifications, acetylation of α-tubulin enhanced intracellular transport efficiency by facilitating interaction with kinesins, thereby accelerating the translocation of synaptic vesicles and mitochondria in neurons [91]. Beyond acetylation, detyrosination of tubulin influences the motility and processivity of specific motors [90, 92, 93] where oligoglutamylation and polyglycylation differentially impact kinesin and dynein movement [90].

Overall, microtubule dynamic adaptations to mechanical cues can impact on the control of cell shape, either directly, by generating or resisting forces, or indirectly, through the modulation of actomyosin contractility, on intracellular organization, and on cell-cell and cell-substrate adhesion. All these modifications can contribute to the maintenance of the integrity of the endothelial barrier. More specifically, microtubules are known to play an essential role in regulating endothelial permeability by interacting with the junctional adhesive proteins and the associated actin cytoskeleton [94, 95]. A key signaling molecule of this interactive unit is GEF-H1, the Rho-specific activator whose activity is regulated by its retention on microtubules. The release and activation of GEF-H1 promote actomyosin contractility, leading to cell retraction and junction destabilization [39, 96]. Alterations in microtubule dynamics at the cell periphery, or even their depolymerization, appear to be critical triggers for barrier disruption in various contexts, including shear stress, whereas a pool of stable microtubules is thought to support endothelial barrier integrity [95]. Thus, changes in microtubule end dynamics, stability or PTMs, triggered by mechanical forces, particularly those promoting microtubule disassembly, can significantly influence endothelial permeability via signaling crosstalk with EC junctions and actin cytoskeleton.

## Role of microtubules in sensing different environmental constraints

### Shear stress

Shear stress, the frictional force exerted by blood flow on ECs, is a key regulator of vascular function. It influences endothelial morphology, gene expression, and intracellular signaling, contributing to vascular remodeling, inflammation, and atherogenesis [3, 5, 97]. The ability of ECs to sense and respond to different shear stress characteristics (magnitude, pulsatility, and direction) is essential for maintaining vascular homeostasis. Under normal physiological conditions, laminar shear stress promotes an anti-inflammatory state, whereas disturbed shear stress, commonly observed at vessel bifurcations and curvatures, sustains inflammatory pathways activation, leading to endothelial dysfunction and early-stage atherosclerosis [4, 98–100]. ECs in culture respond to shear stress by elongating and aligning along the flow axis, a process driven by cytoskeletal reorganization [101] and which resembles the behavior found in vivo [102]. In blood vessels, zones where ECs are elongated and aligned in the direction of blood flow tend to be protected from atherosclerosis [103, 104]. Shear stress induces complex remodeling processes that involve both actin filaments and microtubules [25, 105–107]. While actin filaments are well-established mediators of this response, the role of microtubules in endothelial mechanosensing has received less attention. In the 1990 s, researchers investigating endothelial shape changes under shear stress identified the microtubule network as a structural regulator coordinating endothelial adaptation [57]. Using bovine aortic ECs exposed to physiological shear stress (20 dyn/cm²), their experiments demonstrated that microtubule integrity is essential for actin stress fiber formation and cell elongation. Disrupting microtubules with nocodazole abolished shear stress-induced morphological changes and actin reorganization, while microtubule hyper stabilization with taxol attenuated shape alterations without preventing actin fiber formation. The study also highlighted the roles of intracellular calcium and tyrosine kinases, suggesting that microtubules coordinate with calcium-dependent signaling to mediate endothelial responses to fluid forces [57]. Building on these findings, research in the 2000 s expanded our understanding of microtubule-mediated mechanotransduction under shear stress conditions. McCue et al. confirmed that the dynamic nature of microtubules was required for flow-induced elongation of porcine aortic ECs [41]. Laminar shear stress was also shown to redistribute the centrosome and the microtubules to the downstream side of the nucleus and to promote microtubule acetylation [41]. Expanding on these observations, a recent study has characterized the PTMs of microtubules in response to shear stress and their importance in EC adaptation to hemodynamic forces [25]. The researchers first confirmed the flow-dependent increase in microtubule acetylation, an event occurring very early in the process of cell elongation. Using pharmacological and genetic perturbations of the enzymes required for microtubule acetylation and deacetylation (α-Tat1 and HDAC6), the study further demonstrated that EC elongation and alignment in response to laminar shear stress requires microtubule acetylation. Mechanistically, the increase in acetylation was attributed to the rapid destabilization of the HDAC6 protein. Interestingly, while it artificially increased microtubule acetylation to levels induced by shear stress, hyper stabilization of microtubules using drugs prevented cell elongation and alignment, indicating that both microtubule acetylation and dynamic remodeling are essential for endothelial adaptation to fluid flow [25]. In cancer cells, microtubule acetylation, albeit responding in an opposite manner, was also shown to regulate cellular response to shear stress. In these cells, microtubules undergo deacetylation upon shear stress, which is associated with enhanced caveolin-dependent integrin β1 internalization, ultimately promoting cancer cell migration. The distinct response in acetylation levels may be attributable to the lower shear stress applied in these experiments (1.8 dyn/cm²). Inhibiting HDAC6 blocks these effects, confirming microtubule deacetylation as a key regulator of the mechanism by which shear stress influences cell behavior [108].

### Stretch

Among the continuous dynamic changes that blood vessels experience, mechanical stretch, coming from the pulsatile blood flow and from vessel wall dilation and constriction, emerges as a particularly influential factor, directly impacting the cytoskeletal organization, including microtubules. Recent studies revealed that stretch not only affects EC morphology [109, 110] but also directly modulates microtubule dynamics. In vitro experiments have shown that microtubules align with the transient stretch direction on extensible substrates [59] and that their growth is promoted upon tension applied using laser-based tools as optical tweezers [60]. Mathematical models suggest that tension stabilizes protofilament alignment, favoring microtubule elongation [63].

The regulatory role of stretching forces on microtubule properties has been extended in living cells. In retinal pigment epithelium cells, microtubules respond to cyclic mechanical stretches by being less dynamic, more curved and more stable. Importantly, microtubule stabilization did not relate here to an increase in microtubule PTMs as acetylation, in fact PTMs were unaffected after stretch, but rather to their ability to resist to drug-induced depolymerization. Upon stretching forces, the microtubule-associated protein CLASP relocates from microtubule ends to their shaft, especially to bent regions, preventing depolymerization and explaining the increased stability [24]. Interestingly, this mechano-stabilization occurs independently of the actin- and nucleo-cytoskeleton, highlighting an autonomous role for microtubules in cellular adaptation to forces and establishing microtubules as mechano-responsive elements [24]. In this epithelial context, microtubule stabilization was shown to enhance cell migration in confined spaces. Whereas this highlights the potential relevance of translating stretch-induced microtubule adaptation to ECs, studies on cytoskeletal remodeling following mechanical stretch have, as in many contexts, so far focused primarily on actin [111, 112].

In addition to their potential intrinsic role in mechanotransduction, microtubules interact with actin filaments, a key area of ongoing investigation. For instance, a recent study examined how actin dynamics and anisotropic tension regulate cellular mechanics in endothelial tubes subjected to a physiological increase in luminal pressure using a microstretcher. The study demonstrated that this tensile stress induced shape reorientation and viscoelastic behavior in ECs, relying on actin stabilization through focal adhesions and adherens junctions. However, the complexity of the endothelial mechanical responses, particularly following actin depolymerization, suggests the involvement of other cytoskeletal components. Microtubules are likely contributors, though their specific function remains unexplored.

One context in which the role of microtubules has been specifically investigated is the destabilization of the endothelial lung barrier caused by mechanical stretching from ventilation. While microtubule stability is known to play a pivotal role in regulating barrier integrity [94] cyclic stretch has been shown to induce α-tubulin deacetylation, actin stress fiber formation, and endothelial hyperpermeability [50]. Once again, microtubule (de)acetylation was believed to influence microtubule (in)stability. Notably and in contrast to the situation in shear stress [25] hyper stabilization of microtubules with Taxol in this context rescued the phenotype of hypermeability [50].

### Stiffness

Stiffness is the ability of a material to resist deformation when subjected to an external force, commonly quantified by the elastic modulus (Young’s modulus) in biological and biomechanical contexts. In tissues, stiffness is not uniform but varies depending on physiological and pathological conditions. Pathological conditions such as vascular injury and tumor formation significantly reshape the extracellular microenvironment, directly influencing cellular behaviors like proliferation and migration [13, 113, 114]. Numerous studies have shown that substrate rigidity impacts EC morphology, leading to altered adhesion organization and reinforced actomyosin contraction [23, 115]. Contrary to the prevailing notion that mechanosensitivity is primarily governed by actomyosin and focal adhesions, a recent study reveals that microtubules play a crucial role in substrate rigidity sensing, directly influencing cytoskeletal organization, force transmission, and cell migration. Specifically, the findings, obtained in astrocytes and ECs, demonstrate that substrate rigidity regulates microtubule acetylation via β1 integrin and Talin, which in turn modulates force transmission and activates YAP, the key mechanosensitive transcription factor [23]. Another recent study shows the causal role of microtubule acetylation in mechanosensing and mechanotransduction during collective cell migration of neural crest cells, a process that shares several similarities with the migration of ECs [52]. Using *Xenopus laevis* development as an in vivo model, it was shown that cells reduce their stiffness to initiate migration in response to the progressive stiffening of their substrate. This process is mediated by the mechanosensitive ion channel Piezo1, which triggers microtubule deacetylation. A reduction in α-tubulin acetylation promotes cellular softening [116] a crucial step for the onset of collective migration. In another context, in cancer cells, matrix stiffening induces microtubule glutamylation and stabilization, thereby promoting cell invasion [117]. While differences in cell type, the nature of the mechanical environment, and the mode of migration (collective versus individual), likely contribute to the observed contrasting results, the effect of stiffness on microtubule properties cannot be strictly paralleled. In the case of cellular softening in the neural crest, the associated reduction in microtubule acetylation has not been correlated with their stability. In contrast, in tumor cells, microtubule glutamylation, triggered by metabolism rewiring, has been linked to decreased end dynamics and increased resistance to depolymerization. Notably, microtubule acetylation has not been assessed in this context.

Besides their acetylation, microtubule dynamics can also be modified, globally and locally, by the stiffness of the extracellular matrix, what participates in endothelial responses to guide vascular morphogenesis in complex environments [45]. Extracellular rigidity influences the speed and persistence of microtubule growth through myosin II contractility. A softer extracellular matrix promotes more dynamic microtubules, with faster growth and reduced persistence, increasing the frequency of cellular branching [45].

In vessels, ECs are continuously exposed to a complex interplay of mechanical stresses to which they should react in an integrated manner. Very few studies have tested this multi-variable aspect, however the concurrent response of cancer cells to two of them, stiffness and topography, has been recently characterized and reveals the influence of stiffness on microtubule organization [118]. When plated on densely spaced oriented nanolines, cells behave differently depending on the mechanical rigidity of the substrate. On soft matrix (2.3 kPa), cells elongate and adopt a polarized morphology, with aligned microtubules stabilizing lamellipodial protrusions. Conversely, on stiff substrate (50 kPa), cells develop circular lamellipodia with more dispersed and disorganized microtubules. Disrupting microtubules with nocodazole or hyper stabilizing them with Taxol disturbs the cell alignment, leading to a more circular cell shape in all conditions, underscoring microtubules as key mediators of substrate-dependent cell architecture [118]. The substrate parameters tested in this study are also relevant to the endothelial microenvironment, potentially indicating a similar role for microtubules in ECs.

### Topography

The basement membrane to which ECs adhere features a structured surface with topographical characteristics at multiple scales [119–121]. The vascular topography consists of an intricate network of intermingled fibers and pores [121]. Numerous studies have demonstrated that cells can actively sense the topography of their environment and adjust their biological response accordingly, a sensing that crucially involves the cytoskeleton [122, 123]. In response to anisotropic topographical features, cells have the tendency to elongate, orient and migrate bidirectionally, following substrate-specific behavioral patterns. The observation that EC monolayers collectively migrate as antiparallel cell streams when placed on anisotropic microgroove substrates [10] is an example of this process called contact guidance [124]. The current model implies that the filopodia, actin-rich extensions, act as primary detectors of nanoscale structures, align along nanogrooves and guide the organization of focal adhesions [125]. These integrin-based adhesions transmit topographical information to the actin network, leading to adjustments in cell migration, morphology, and contractility [126, 127]. Cytoskeletal modifications induced by topography can also reshape nuclear architecture and modulate gene expression by transmitting forces to the nucleus via the LINC complex [128]. Thus, by linking the extracellular environment to intracellular responses, the cytoskeleton emerges as a key player in topographical sensing and cellular adaptation. Until lately, the role of microtubules in topography sensing remained unexplored and has been limited to their general function as a counterbalancing element to the actin-associated tension, contributing to force transmission and cellular integrity.

A recent study shows that microtubules play an integral function in topography sensing, particularly in EC elongation on microgrooved substrates that mimic the topography of the thin fibers of the underlying extracellular matrix [46]. Contrary to expectations, actin inhibition does not prevent cell alignment, whereas microtubule disruption significantly reduces elongation, highlighting their essential role in controlling cell morphology on microstructured surfaces.

At a different scale, studies on nanotextured substrates provide further insights into microtubule-mediated topography sensing. On such surfaces, microtubules become sterically trapped within nanogrooves, strengthening cell elongation and alignment along contact guidance cues [118]. Whereas intact microtubules are required for ECs to conform to nanotopographic features, their interaction with actin stress fibers further refines this mechanosensing process. Within nanogrooves, microtubules engage with actin stress fibers, mechanically reinforcing protrusions. This cooperation is finely tuned by cytoskeletal regulators. The balance between Arp2/3-driven lamellipodia spreading and Formin-dependent stress fibers formation regulates microtubule positioning, ensuring effective topographic sensing and guided migration [118].

### Curvature

The influence of curvature on cellular behavior has only recently been explored and is now recognized as a key factor in regulating cell responses [14]. ECs in the vasculature are exposed to curvature at multiple levels, ranging from the subcellular to the tissue scale. The actin cytoskeleton very recently emerged as a central player in the curvature-induced response. Substrate curvature significantly influences EC alignment, morphology [11] and collective cell migration [129, 130]. Cells preferentially align along concave microgrooves with a 50 μm radius, while alignment decreases at larger curvatures and disappears on flat surfaces. This alignment is closely linked to the organization of stress fibers as their inhibition with blebbistatin reduces cell aspect ratio and abolishes curvature-induced alignment [11]. Myosin activity was also involved, together with focal adhesion, in the decrease in directional EC migration speed observed on curved topography [129]. Another study shows that the curvature of hydrogel microgrooves influences EC morphology, orientation, apoptosis, and traction force, with a strong involvement of the actin cytoskeleton and integrins, but still no investigation of the role of microtubules [131]. Curvature is also studied by culturing cells on fibers [132]. One study identifies actin grips, a novel actin cytoskeletal structure that enables ECs to wrap around polymeric microfibers [133]. The formation of these specific actin structures depends on fiber curvature, suggesting that cells adapt their cytoskeletal architecture to match the geometry of their substrate.

Cumulative evidence from these studies underscores the critical role of the cytoskeleton in curvature sensing. However, the role of microtubules has not been investigated, despite their known involvement in other forms of mechanosensing, an important gap that deserves more attention.

## Conclusions

**Fig. 5:**
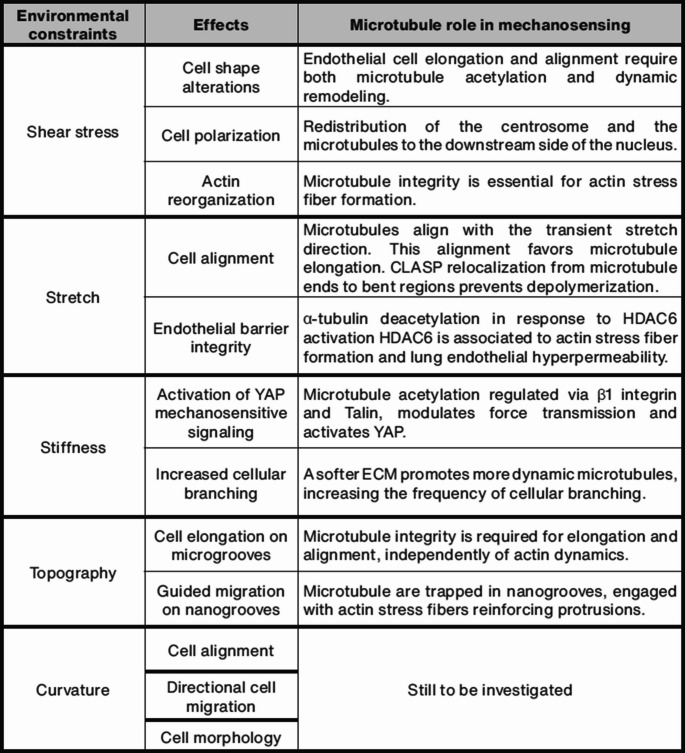
Table recapitulating the role of microtubule rearrangements on the EC responses to mechanical stresses

While the role of microtubules in mechanosensing remains relatively underexplored compared to other cytoskeletal elements, accumulating evidence suggests that they play a crucial role in responding to mechanical cues, including in the context of blood vessels (Fig. 5). The fact that endothelial microtubules are profoundly and dynamically modified in response to mechanical stress underscores their potential as key regulators of mechanotransduction. Due to their various properties, microtubules can participate in the initial perception of mechanical stimuli, transmit the signal to downstream effectors, but also contribute to cellular shape modulation, either directly or through their interactions with other cytoskeletal components.

Several studies have pointed to a role for microtubule integrity in the EC response to various mechanical stresses. However, the widespread use of pharmacological agents such as Taxol and nocodazole to study microtubule function presents challenges due to their unintended effects: Taxol causes massive microtubule acetylation and cellular contractility is increased in response to microtubule depolymerization triggered by nocodazole. This complicates the interpretation of results and highlights the need for alternative approaches to dissect the specific contributions of microtubule integrity and dynamics in mechanosensing. Intriguingly, emerging studies emphasize the significance of tubulin acetylation and the enzymes responsible for its regulation in response to mechanical stimuli. Investigating the upstream regulation of these enzymes could provide critical insights into the precise mechanisms governing microtubule-mediated EC mechanotransduction. Additionally, MAPs serve as essential regulators of microtubule behavior and function, yet their potential role in mechano-sensitive recruitment and response to distinct types of mechanical stress remain largely unexplored.

To overcome the limitations associated with classical pharmacological agents such as nocodazole or Taxol, future research should leverage advanced tools to dissect the response and the role of microtubules in endothelial mechanosensing. As a first step, a systematic assessment of microtubule properties under mechanical stress is essential. This includes quantifying microtubule mass, evaluating their resistance to depolymerization, characterizing plus-end dynamics, and profiling PTMs. This will enable more meaningful comparisons across studies and conditions. Then, functional interrogation of how microtubules participate in endothelial cell mechanosensing should increasingly rely on genetic tools. Targeted manipulation of enzymes that regulate microtubule PTMs but also of proteins that govern microtubule growth, shrinkage or stability may help isolate the effects of altered microtubule properties in mechanotransduction. Optogenetic strategies could additionally enable precise spatiotemporal control over the activity of these proteins [134, 135]. Regarding PTMs and more specifically acetylation, live-cell imaging approaches using live fluorescent tools [136–138] could help visualize dynamic changes in response to mechanical stimuli. In parallel, building PTM-mimetic or -deficient mutant cells using CRISPR-mediated knock-in strategies would provide a powerful approach to directly assess the functional contribution of specific modifications in a physiological context. Future research should also aim to integrate multiple forms of mechanical stress to better capture the complexity of vascular environments and their impact on endothelial microtubules.

Given the strong link between microtubules, angiogenesis, and pathological conditions, further investigation into their mechanosensitive properties could yield significant therapeutic implications. More specifically, microtubules may represent promising intervention targets in diseases driven by mechanical stress, such as cancer, atherosclerosis, and fibrosis. These conditions are characterized by an altered mechanical environment that reshapes cell behavior through cytoskeletal remodeling. As discussed throughout this review, microtubules not only adapt to mechanical cues via PTMs, association with MAPs or changes in their dynamics, but also actively mediate mechanosensing, signaling, and structural adaptation.

In atherosclerosis, endothelial dysfunction is exacerbated by exposure to disturbed shear stress and is associated with impaired EC elongation and alignment, compromising vascular integrity. Since ECs rely on microtubules for proper cellular responses to shear stress, targeting them may help restore endothelial responsiveness and protect vascular function, particularly under disturbed shear stress conditions. Notably, microtubule stability and/or acetylation have been shown to be essential for endothelial adaptations to shear stress. Therefore, the use of microtubule-stabilizing drug, as Taxol, or pharmaceutical modulation of acetylation regulators such as αTAT1 or HDAC6, could hold therapeutic potential. Of note, Taxol has demonstrated anti-atherogenic effects in rabbit models, although these effects were originally attributed to its antiproliferative activity on smooth muscle cells [139].

In addition to the prominent role of fibroblasts, ECs have emerged as key contributors to fibrosis and tissue remodeling, either directly by transdifferentiating into myofibroblasts through processes of transient mesenchymal activation, or indirectly through the secretion of angiocrine profibrotic and proinflammatory mediators [140, 141]. Activation of the ECs is probably driven by several cytokines and growth factors, but other putative mechanisms include matrix stiffening, which is commonly observed during fibrosis [141]. While changes in tubulin isotype expression, potentially impacting on microtubule dynamics and stability, have been documented in cellular models of endothelial-mesenchymal transition during fibrosis [142] matrix stiffening alters microtubule dynamics and PTMs, as described above, thereby affecting cell mechanics and cell morphology. Pharmacologically targeting these microtubule-related modifications may thus help counteract fibrotic progression by limiting EC activation and morphological changes.

Tumor vasculature, the network of blood vessels that supplies tumors and supports their growth, is a key factor in cancer progression and a long-standing target for therapy, either via disruption or normalization of these vessels [143]. Indeed, unlike the organized vasculature in healthy tissue, tumor blood vessels are structurally and functionally abnormal, with irregular shapes, leaky walls, and poor blood flow. These abnormalities result not only from biochemical signals but also from mechanical forces such as compression stresses due to tumor expansion, elevated interstitial fluid pressure, and altered tissue stiffness in the tumor microenvironment [144]. Microtubule-targeting agents such as Taxol or vinka alkaloids are already widely used in cancer treatment, where they prevent cell proliferation but have also shown anti-angiogenic effects, reinforcing their relevance in oncology. A better understanding of the role of microtubules and their properties in the endothelial mechanical response within the tumoral context could inform the development of more targeted therapies and help remediate current challenges, such as systemic toxicity.

Overall, microtubules represent a promising yet underappreciated component of the cellular mechanosensing machinery, warranting deeper exploration to fully uncover their regulatory functions in vascular biology.

## Data Availability

Not applicable.

## Glossary

ACF7: Actin Crosslinking Family 7, a protein involved in microtubule-actin interactions.
α-TAT1: Alpha-tubulin acetyltransferase 1, an enzyme responsible for the acetylation of α-tubulin.
APC: Adenomatous Polyposis Coli, a tumor suppressor protein that regulates microtubule dynamics and microtubule-actin interactions.
Arp2/3: Actin-related protein 2/3 complex, crucial for actin nucleation and branched actin filament formation.
Blebbistatin: A small molecule inhibitor of myosin II, often used to study the role of actomyosin contractility.
Centrosome: The primary microtubule-organizing center of the cell, essential for mitotic spindle formation.
CLASP: Cytoplasmic linker-associated proteins, involved in microtubule stabilization.
Dynamic instability: Specific behavior of microtubule plus-ends that alternate between phases of growth, shortening, and pause.
Dynein: A motor protein that moves along microtubules towards the minus-end, crucial for intracellular transport and mitotic spindle positioning.
Formin: A protein family involved in actin filament elongation and regulation.
GEF-H1: A guanine nucleotide exchange factor (GEF) that activates RhoA, linking microtubule dynamics to actin cytoskeleton regulation.
GSK-3β: Glycogen synthase kinase 3 beta, a kinase involved in multiple signaling pathways, including microtubule regulation.
HDAC6: Histone deacetylase 6, an enzyme involved in microtubule deacetylation.
Integrin: A transmembrane receptor involved in cell adhesion, migration, and signal transduction.
KASH: Klarsicht, ANC-1, and Syne homology domain proteins, outer nuclear membrane part of the LINC complex.
Kinesin: A microtubule-associated motor protein that moves towards the plus-end, responsible for intracellular transport.
LINC: LInker of Nucleoskeleton and Cytoskeleton complex, connecting the cytoskeleton to the nuclear envelope.
MAP: Microtubule-associated proteins, a group of proteins that associate with and regulate microtubules.
Myosin: A motor protein that interacts with actin filaments, playing a role in cell contraction.
Nesprin: Nuclear envelope spectrin-repeat proteins, part of the LINC complex that links the nucleus to the cytoskeleton.
Nocodazole: A drug that stimulates microtubule depolymerization.
PCP (Planar Cell Polarity): A signaling pathway that regulates the coordinated orientation of cells within a tissue plane.
Piezo1: A mechanosensitive ion channel involved in cellular responses to mechanical stimuli.
Rab6: A small GTPase involved in Golgi-to-ER, intra-Golgi and Golgi to the plasma membrane transport.
RhoA: A small GTPase regulating the actin cytoskeleton, cell contractility, and migration.
ROCK: Rho-associated protein kinase, a downstream effector of RhoA involved in actin-myosin contractility.
SUN1: A nuclear envelope protein that interacts with KASH-domain proteins in the LINC complex.
TAK1: Transforming growth factor-beta-activated kinase 1, involved in inflammatory and stress signaling pathways.
Talin: A mechano-effector cytoskeletal protein linking integrins to the actin cytoskeleton.
Taxol: A drug that stabilizes microtubules and reduces their dynamics.
Tau: A microtubule-associated protein involved in stabilizing microtubules, particularly in neurons.
TGF-β: Transforming growth factor-beta, a signaling molecule regulating cell growth, differentiation, and migration.
VEGF: Vascular endothelial growth factor, a key regulator of angiogenesis.
YAP: Yes-associated protein, a transcriptional co-activator involved in mechanotransduction and Hippo signaling.
